# Code Status Transitions of Patients with Aneurysmal Subarachnoid Hemorrhage in the Intensive Care Unit

**DOI:** 10.1089/pmr.2025.0015

**Published:** 2025-06-04

**Authors:** Min-I Su, Chia-Ying Hsiao, Jui-Chu Ma, Che-Ming Chang

**Affiliations:** ^1^ Department of Medicine, MacKay Medical College, New Taipei, Taiwan.; ^2^ Department of Internal Medicine, Division of Cardiology, Taitung MacKay Memorial Hospital, Taitung, Taiwan.; ^3^ Department of Internal Medicine, Division of Nephrology, Taitung MacKay Memorial Hospital, Taitung, Taiwan.; ^4^ Department of Nursing, Taitung Mackay Memorial Hospital, Taitung, Taiwan.; ^5^ Department of Nursing, Tamsui Mackay Memorial Hospital, New Taipei, Taiwan.; ^6^ Nursing, and Management Guandu Campus, Mackay Junior College of Medicine, New Taipei, Taiwan.

**Keywords:** aneurysmal subarachnoid hemorrhage, code status transition, do-not-intubate, do-not-resuscitate

## Abstract

**Background:**

Aneurysmal subarachnoid hemorrhage (aSAH) carries high mortality rates and often requires critical family decisions about code status when complications occur. The American Heart Association provides treatment guidelines but acknowledges a significant knowledge gap regarding do-not-resuscitate or do-not-intubate (DNR/DNI) decisions in patients with aSAH, challenging clinicians in identifying appropriate timing for these discussions.

**Aim:**

To identify demographic and clinical physiological factors associated with code status transition in adults with aSAH admitted to the intensive care unit, supporting value-based decision making through more informed and timely discussions between health care providers and families that align with patients’ core values and preferences.

**Methods:**

Retrospective cohort study analyzing Medical Information Mart for Intensive Care IV database (2008–2022) data from 731 patients with aSAH. Researchers collected demographics, vital signs, laboratory tests, disease severity scores, and code status transition, performing univariate and multivariate Cox regression analyses to identify significant predictors.

**Results:**

Among patients initially with full-code status, 25.8% transitioned to DNR/DNI during hospitalization. Multivariate analysis identified four independent predictors: advanced age (hazard ratio [HR] = 1.024), lower mean blood pressure (HR = 0.987), higher simplified acute physiology score II (SAPS II) score (HR = 1.018, each one-point increase raises transition risk by 1.8%), and hospice services (HR = 6.951). Patients with code status limitations received less invasive therapy, more hospice services, and had higher mortality rates.

**Conclusion:**

Age, blood pressure, SAPS II, and hospice services predict code status transitions in patients with aSAH. Identifying high-risk patients enables timely code status discussions, ensuring treatment aligns with patient values and improving family decision making during critical situations.

## Introduction

Cerebral aneurysms are thin-walled protrusions of intracranial arteries that may rupture, causing subarachnoid hemorrhage (SAH). Aneurysmal SAH (aSAH) presents as a life-threatening condition with significant mortality (26% of patients succumb prior to hospital presentation)^1^ and morbidity (8.09 million individuals were diagnosed with this condition globally in 2023).^2^ Despite variations in management approaches across health care systems internationally, most follow similar principles based on guidelines such as those from the American Heart Association (AHA), which recommend initial management that includes securing the ruptured aneurysm via endovascular coiling or surgical clipping, treating hydrocephalus with external ventricular drainage (EVD), and subsequent intensive care unit (ICU) admission for comprehensive monitoring and management of complications.^3^

Severe acute complications (e.g., rebleeding, acute cerebral injury, acute hydrocephalus, elevated intracranial pressure) lead to in-hospital mortality rates as high as 13.9%−20%.^3,4^ When these complications occur, family members must make critical decisions regarding secondary interventions within a limited time. Existing research mainly focuses on long-term survival rates,^5^ functional prognosis prediction,^6^ or acute mortality^7^ of patients with aSAH. However, previous studies have shown that aSAH survivors often experience permanent neurological deficits, severely affecting functional outcomes and quality of life.^8^ Additionally, aSAH frequently affects middle-aged individuals who typically lack advance medical directives,^9^ creating significant challenges for family members facing critical decisions.

In clinical practice, code status discussions regarding do-not-resuscitate or do-not-intubate (DNR/DNI) are particularly critical and complex for patients with aSAH. AHA care guidelines clearly identify the existing knowledge gaps regarding acute resuscitation and early DNR orders in patients with aSAH,^3^ indicating that health care providers lack clear guidance on when and based on which clinical indicators to initiate such discussions, while family members must make critical decisions concerning quality of life and dignity within an extremely short timeframe when acute complications occur.^10^ Despite these challenges, existing research largely focuses on parameters for predicting general prognostic or mortality,^11,12^ rather than specifically investigating factors influencing code status decisions, resulting in a lack of sufficient research in clinical practice to understand which parameters may be key factors influencing family members’ willingness to transition code status.

This represents a significant gap in literature with important clinical implications. By identifying key factors associated with code status transition, this study provides physiological indicators that clinical teams can use to identify patients with aSAH needing early code status discussions, an important contribution not yet provided by existing research. Early involvement in care preference discussions can improve the quality of life for patients and families, reduce caregiver distress, and help achieve consistent care goals.^13^ Therefore, this study aims to identify demographic and clinical physiological factors associated with code status transition in adults with aSAH admitted to the ICU, providing insights to support more informed and timely discussions between health care providers and families.

## Methods

### Data source and population

The cohort study data were obtained from 2008 to 2022, from the Medical Information Mart for Intensive Care IV (MIMIC-IV version 3.1) database. This is a publicly accessible, real-world clinical database managed by the Beth Israel Deaconess Medical Center (BIDMC) in Boston, Massachusetts, a teaching hospital of Harvard Medical School and one of the founding members of Beth Israel Lahey Health. The database includes data from different types of intensive care units within this single academic medical center, encompassing over 200,000 emergency department admissions and more than 60,000 ICU stays.^14,15^ It offers comprehensive data for each patient, such as laboratory tests, vital signs, medication administration, and length of stay. One of the authors, M.-I.S., who completed the Collaborative Institutional Training Initiative examination (certification number: 42219644), obtained permission to access the database and was responsible for data extraction using the code available on GitHub (https://github.com/MIT-LCP/mimic-iv). The compilation of patient data and the creation of the research asset were reviewed and approved by the Institutional Review Board of the BIDMC, which waived the requirement for informed consent and authorized the data-sharing initiative.^16^

### Eligibility criteria

**FIG. 1.**
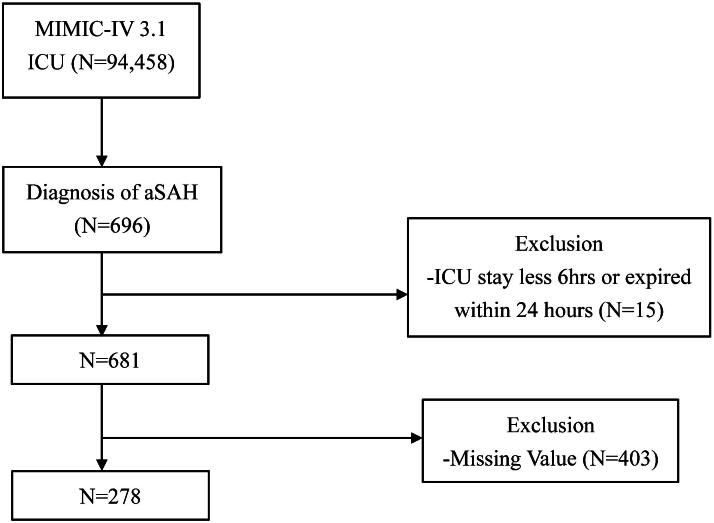
Flow diagram of study participants.

This study included adult patients (>18 years) admitted to the ICU with aSAH. The diagnosis of aSAH was confirmed using the International Classification of Diseases (ICD) codes, specifically the ICD-9-CM: 437.3, 430; ICD-10-CM: I67.1, I60. For patients with multiple ICU admissions, only the data from their first ICU admission were included. We also excluded missing data (incomplete vital signs or laboratory tests), no code status order, and patients who had an ICU stay of <24 hours, as such short stays may not reflect a full critical care episode or provide adequate clinical data for a meaningful analysis (Fig. 1).

### Data extraction and processing

Data extracted within 24-hour window around ICU admission included demographics (gender, age),^17^ vital signs (heart rate, mean blood pressure [MBP], respiratory rate, temperature, and peripheral oxygen saturation [SP $O_{2}$]),^17^ laboratory tests (glucose, sodium, potassium, blood urea nitrogen [BUN], creatinine, international normalized ratio [INR], hemoglobin, platelets, white blood cell count, red blood cell count [RBC]),^17^ Glasgow Coma Scale (GCS),^17^ disease severity assessment tools (Charlson comorbidity index [CCI], Sequential Organ Failure Assessment [SOFA], and the simplified acute physiology score II [SAPS II]).^18,19^ Invasive therapy (refers to endovascular treatment or surgical treatment for intracranial aneurysms using the following related codes: ICD-9-PCS: 3972, 3975, 3976, 3951, 3952, and ICD-10-PCS: 03V[QPLKG]3[BD]Z, 03L[QPLKG]3[BD]Z, 03V[QPLKG]0[PCDZ]). We also collected data on ICU admission via the emergency department, hospice services (defined as patients who newly established contact with, consultation for, or referral to hospice care during their hospital stay, as identified through discharge codes in the database: ICD-9-PCS: V667, ICD-10-PCS: Z51.5), ICU stay and hospital stay (Table 1).

**Table 1.** Characteristics and Outcomes of Aneurysmal Subarachnoid Hemorrhage Patients by Code Status during the 24-Hour Period around Intensive Care Unit Admission

		Code status		
	All patient	Limitation group	Full code group	
Mean (SD)/*n*(%)	(*N* = 731)	(*N* = 164)	(*N* = 567)	*p*-Value
Gender (male)	339 (46.37)	66 (40.24)	273 (48.15)	0.044^*^
Age	63.15 (15.29)	70.08 (14.96)	61.14 (14.81)	0.000***
Heart rate (bpm)	83.55 (18.94)	83.41 (19.63)	83.59 (18.75)	0.917
MBP (mmHg)	88.09 (17.54)	86.15 (18.36)	88.66 (17.27)	0.106
Respiratory rate (insp/min)	18.83 (5.34)	19.47 (4.92)	18.65 (5.45)	0.084
Temperature (°C)	36.76 (0.92)	36.63 (1.13)	36.8 (0.84)	0.087
SP $O_{2}$ (%)	97.59 (3.14)	97.98 (2.68)	97.48 (3.26)	0.044^*^
Glucose (mg/dL)	154.33 (66.02)	159.56 (59.99)	152.81 (67.64)	0.249
Sodium (mEq/L)	138.83 (4.93)	139.27 (5.37)	138.7 (4.79)	0.188
Potassium (mEq/L)	4.07 (0.79)	4.06 (0.89)	4.08 (0.76)	0.861
BUN (mg/dL)	20.23 (15.78)	21.78 (14.34)	19.78 (16.16)	0.153
Creatinine (mg/dL)	1.08 (1.02)	1.06 (0.62)	1.09 (1.11)	0.756
INR	1.27 (0.63)	1.26 (0.47)	1.27 (0.67)	0.914
Hemoglobin (g/dL)	12.19 (2.44)	12.19 (2.4)	12.2 (2.45)	0.982
Platelet (K/μL)	223.85 (109.93)	221.24 (123.99)	224.6 (105.63)	0.731
WBC (K/μL)	13.16 (11.17)	14.15 (6.67)	12.87 (12.15)	0.196
RBC (m/μL)	4.03 (0.81)	4.06 (0.84)	4.02 (0.8)	0.657
GCS	12.56 (3.58)	11.89 (4.18)	12.76 (3.37)	0.015^*^
CCI	4.71 (2.67)	5.43 (2.79)	4.5 (2.6)	0.000***
SOFA	4.25 (3.54)	4.81 (3.26)	4.09 (3.6)	0.021^*^
SAPS II	35.66 (14.54)	43.29 (12.96)	33.45 (14.24)	0.000***
Invasive therapy (yes/no)	255 (34.88)	37 (22.56)	218 (38.45)	0.000***
Hospice services (yes/no)	160 (21.89)	77 (46.95)	83 (14.64)	0.000***
Admission via emergency department (yes/no)	332 (45.42)	73 (44.51)	259 (45.68)	0.859
ICU stay (day)	8.2 (9.22)	6.38 (7.57)	8.72 (9.59)	0.001**
Hospital stay (day)	15.68 (19.22)	9.31 (11.38)	17.52 (20.59)	0.000***
DNR	164 (22.44)	164 (100)	0 (0)	0.000***
DNI	99 (13.54)	99 (60.37)	0 (0)	0.000***
ICU mortality	170 (23.26)	94 (57.32)	76 (13.4)	0.000***
In-hospital mortality	220 (30.1)	117 (71.34)	103 (18.17)	0.000***

^*^p < 0.05; ^**^p < 0.01; ^***^p < 0.001.

BUN, blood urea nitrogen; CCI, Charlson comorbidity index; DNI, do-not-intubate; DNR, do-not-resuscitate; GCS, Glasgow Coma Scale; ICU, intensive care unit; INR, international normalized ratio; MBP, mean blood pressure; RBC, red blood cell count; SAPS II, the simplified acute physiology score II; SD, standard deviation; SOFA, Sequential Organ Failure Assessment; SPO_2_, peripheral oxygen saturation; WBC, white blood cell count.

### Code status transition

We reviewed the electronic health records (EHR) from the MIMIC-IV database to identify code status transitions among patients with aSAH in the ICU. Code status was categorized into two groups: full code group (no code limitations) and limitation group (DNR or DNI). We collected the dates and times of code status documentation and analyzed two critical time points: the initial code status documented within the first 24 hours of ICU admission and subsequent code status transitions during the ICU-to-discharge period.

### Processes leading to code status order transition from full code to DNR

Two researchers (M.-I.S. and C.-Y.H.) independently reviewed the EHR of the patients diagnosed with aSAH in the MIMIC-IV database and documented the processes of code status transitions from full code to DNR or DNR/DNI. The researchers conducted detailed examinations of the MIMIC-IV database, searching for key terms such as “code status,” “DNR,” “DNI,” and “full code” to ensure a comprehensive collection of the data related to code status transitions. Similar methodology has previously been employed to examine code status transitions among patients who have experienced an acute ischemic stroke.^20^

### Statistical analysis

Continuous variables were described using means and standard deviations, while categorical variables were presented as frequencies and percentages. For between-group comparisons, t-tests were used for continuous variables and chi-square tests for categorical variables, with a significance level of *p* < 0.05. To identify factors associated with code status transition, we conducted Cox proportional hazards regression analysis. We first performed univariate analysis to identify statistically significant variables, then incorporated these variables into a multivariate Cox regression model to determine independently associated predictors. Results were expressed as hazard ratios (HR) with corresponding 95% confidence intervals (CI), with statistical significance defined as *p* < 0.05.

## Results

Our analysis included 731 patients with aSAH. Among these patients, 567 (77.56%) had full code status and 164 (22.44%) had code status limitations (DNR/DNI) during the first 24 hours after ICU admission. Patients with early code status limitations were significantly older, had a lower proportion of males, and demonstrated lower GCS and higher CCI, SOFA, and SAPS II (all *p* < 0.05).

Follow-up outcome analysis showed that patients with early code status limitations received less invasive therapy and higher rates of hospice services. They also had shorter ICU stays and overall hospital lengths of stay. Mortality outcomes were significantly higher in the early code status limitation group, with elevated ICU mortality and in-hospital mortality rates (Table 1).

Among the 567 patients initially with full code status, 146 (25.8%) transitioned to DNR/DNI during hospitalization. These patients were significantly older, predominantly male, and had different vital signs including higher heart rate, higher respiratory rate, and lower MBP. Laboratory tests also differed, with higher glucose, BUN, creatinine, and INR, as well as lower hemoglobin, platelet, and RBC count. The code status transition group demonstrated greater illness severity with higher CCI, SOFA, and SAPS II. They received less invasive therapy (23.29% vs. 43.71%), more hospice services (52.05% vs. 1.66%), and experienced significantly higher mortality rates across all measures (ICU and in-hospital mortality) (Table 2).

**Table 2.** Characteristics and Outcomes of Aneurysmal Subarachnoid Hemorrhage Patients with Code Status Transition during Intensive Care Unit-to-Discharge Period

	Full-code transition		*p*-Value
*N* = 567	No transition	Transition	
Mean (SD)/*n* (%)	(*N* = 421)	(*N* = 146)	
Gender (Male)	189 (44.89)	84 (57.53)	0.006**
Age	59.03 (14.77)	67.23 (13.2)	0.000***
Heart rate (bpm)	81.89 (18.34)	88.51 (19.13)	0.000***
MBP (mmHg)	89.79 (17.62)	85.39 (15.83)	0.008**
Respiratory rate (insp/min)	17.95 (4.97)	20.66 (6.23)	0.000***
Temperature (°C)	36.76 (0.83)	36.9 (0.87)	0.076
SP $O_{2}$ (%)	97.66 (3.07)	96.94 (3.71)	0.035^*^
Glucose (mg/dL)	145.12 (56.58)	175.01 (88.98)	0.000***
Sodium (mEq/L)	138.91 (4.37)	138.09 (5.81)	0.120
Potassium (mEq/L)	4.05 (0.7)	4.15 (0.89)	0.199
BUN (mg/dL)	17.89 (14.43)	25.23 (19.39)	0.000***
Creatinine (mg/dL)	1 (0.94)	1.35 (1.46)	0.009**
INR	1.22 (0.66)	1.41 (0.67)	0.004**
Hemoglobin (g/dL)	12.41 (2.39)	11.57 (2.51)	0.000***
Platelet (K/uL)	235.74 (102.41)	192.46 (108.51)	0.000***
WBC (K/uL)	12.56 (13.21)	13.77 (8.36)	0.303
RBC (m/uL)	4.1 (0.76)	3.81 (0.87)	0.000***
GCS	13.01 (3.03)	12.03 (4.11)	0.009**
CCI	4.02 (2.45)	5.9 (2.52)	0.000***
SOFA	3.44 (3.07)	5.95 (4.32)	0.000***
SAPS II	30.32 (12.82)	42.49 (14.28)	0.000***
Invasive therapy (yes/no)	184 (43.71)	34 (23.29)	0.000***
Hospice services (yes/no)	7 (1.66)	76 (52.05)	0.000***
Admission via emergency department (yes/no)	209 (49.64)	50 (34.25)	0.001**
ICU stay (day)	8 (9.43)	10.81 (9.76)	0.002**
Hospital stay (day)	16.51 (19.41)	20.44 (23.49)	0.047^*^
ICU mortality	13 (3.09)	63 (43.15)	0.000***
In-hospital mortality	15 (3.56)	88 (60.27)	0.000***

^*^p < 0.05; ^**^p < 0.01; ^***^p < 0.001.

Table 3 presents the Cox regression analysis for factors associated with code status transition. In univariate analysis, multiple factors were significantly associated with code status transition, including age, heart rate, MBP, respiratory rate, SPO_2_, glucose, BUN, INR, hemoglobin, platelet, RBC, severity scores (CCI, SOFA, SAPS II), invasive therapy, hospice services, and admission via emergency department.

**Table 3.** Factors Associated with Code Status Transition in Aneurysmal Subarachnoid Hemorrhage Patients: Cox Regression Analysis

	Univariate analysis				Multivariate analysis			
	HR	95% CI		*p*-Value	HR	95% CI		*p*-Value
		Lower	Upper			Lower	Upper	
Gender (male)	0.789	0.566	1.100	0.162	1.073	0.745	1.546	0.706
Age	1.036	1.024	1.049	0.000***	1.024	1.006	1.042	0.008**
Heart rate (bpm)	1.010	1.001	1.018	0.022^*^	1.007	0.996	1.019	0.224
MBP (mmHg)	0.986	0.976	0.997	0.009**	0.987	0.975	0.998	0.023^*^
Respiratory rate (insp/min)	1.052	1.023	1.081	0.000***	1.011	0.977	1.047	0.514
Temperature (°C)	1.114	0.928	1.337	0.245				
SP $O_{2}$ (%)	0.959	0.922	0.999	0.044^*^	0.977	0.928	1.028	0.368
Glucose (mg/dL)	1.003	1.001	1.005	0.001**	1.001	0.999	1.003	0.310
Sodium (mEq/L)	0.971	0.940	1.003	0.077				
Potassium (mEq/L)	1.046	0.854	1.280	0.664				
BUN (mg/dL)	1.012	1.005	1.019	0.001**	0.995	0.985	1.005	0.317
Creatinine (mg/dL)	1.101	0.994	1.220	0.065				
INR	1.146	1.003	1.310	0.045^*^	1.153	0.952	1.395	0.144
Hemoglobin (g/dL)	0.937	0.884	0.992	0.027^*^	1.031	0.876	1.214	0.710
Platelet (K/μL)	0.997	0.996	0.999	0.001**	1.000	0.998	1.002	0.756
WBC (K/μL)	1.004	0.992	1.016	0.502				
RBC (m/μL)	0.815	0.683	0.972	0.023^*^	1.211	0.746	1.966	0.438
GCS	0.964	0.923	1.006	0.092				
CCI	1.175	1.116	1.236	0.000***	0.973	0.896	1.057	0.514
SOFA	1.097	1.055	1.141	0.000***	0.998	0.922	1.080	0.964
SAPS II	1.033	1.023	1.043	0.000***	1.018	1.000	1.036	0.046^*^
Invasive therapy (yes/no)	0.433	0.295	0.638	0.000***	0.715	0.443	1.154	0.170
Hospice services (yes/no)	8.090	5.826	11.235	0.000***	6.951	4.626	10.44	0.000***
Admission via emergency department (yes/no)	0.525	0.371	0.742	0.000***	1.087	0.712	1.658	0.700

^*^p < 0.05; ^**^p < 0.01; ^***^p < 0.001.

The multivariate analysis revealed that after adjusting for confounding variables, four factors remained independently associated with code status transition. Age was significantly associated with code status transition (HR = 1.024, 95% CI: 1.006–1.042, *p* = 0.008), with each additional year increasing the risk by 2.4%. MBP showed an inverse relationship (HR = 0.987, 95% CI: 0.975–0.998, *p* = 0.023), with each 1 mmHg increase associated with a 1.3% decrease in the risk of code status transition. SAPS II, representing overall illness severity, independently predicted code status transition (HR = 1.018, 95% CI: 1.000–1.036, *p* = 0.046). Most notably, hospice service emerged as the strongest independent predictor (HR = 6.951, 95% CI: 4.626–10.446, *p* < 0.001), with patients receiving hospice service having nearly seven times higher occurrence of transitioning to limited code status.

**FIG. 2.**
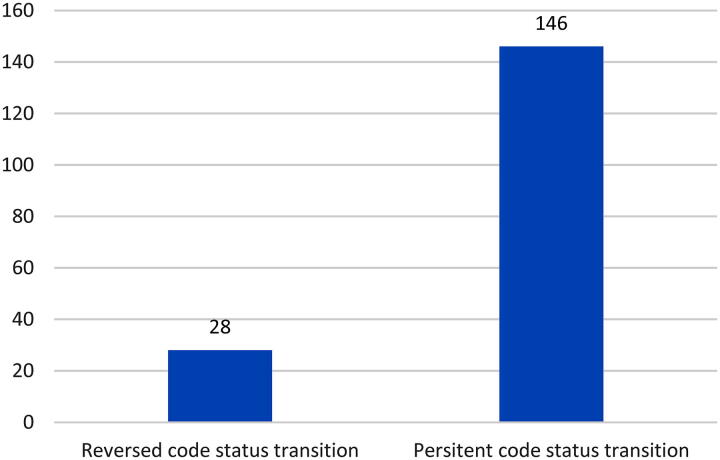
Further analysis of code status patterns in 146 patients who transitioned from full code to limitation: persistent versus reversed.

Figure 2 illustrates the subsequent code status patterns among 146 patients who transitioned from full code to limited code status. Among these patients, we observed two distinct subsequent patterns. The majority (118 patients, 80.8%) maintained their limited code status throughout the remainder of their hospitalization (persistent limitation). However, a subset of patients (28, 19.2%) later reversed their decision, transitioning back to full code status.

## Discussion

This study investigates the determinants of code status transitions in patients diagnosed with aSAH admitted to the ICU. Our findings reveal that among patients with aSAH initially designated as full code, 25.8% experienced a code status transition during hospitalization. Multivariate Cox regression analysis identified four independent predictors: advanced age, lower MBP, higher SAPS II, and hospice services.

Multivariate analysis demonstrated that age was a significant independent predictor of code status transition (HR = 1.024, 95% CI: 1.006–1.042, *p* = 0.008), reflecting its importance as a clinical consideration in decision making for patients with aSAH. Although direct investigations of aSAH and code status transitions are lacking in existing literature, this finding aligns with results from multiple studies examining the relationship between age and DNR or DNI orders.^21,22^ Specifically in neurovascular disease, Soomägi et al. confirmed that advanced age is a key factor in DNR decisions for intracerebral hemorrhage (ICH).^23^ While age is a significant predictor, its relatively modest HR suggests it constitutes one component of multifaceted considerations rather than a sole determining factor. From a clinical practice perspective, age serves as a readily accessible objective parameter that can function as a practical indicator for identifying patients who may require early code status discussions, though health care providers should avoid making judgments based solely on age and instead incorporate it into a comprehensive assessment.

Although multiple vital signs were significantly associated with code status transitions in univariate analysis, only MBP maintained significance in multivariate analysis (HR = 0.987, *p* = 0.023). The finding of MBP as an independent predictor warrants further investigation, especially considering the current lack of consensus in clinical guidelines regarding blood pressure management in aSAH. AHA guidelines only recommend avoiding severe hypotension (mean arterial pressure <65 mmHg) and maintaining systolic blood pressure <160 mmHg or 180 mmHg,^3^ while the Neurocritical Care Society does not provide specific blood pressure recommendations due to insufficient levels of evidence.^24^ For patients with aSAH, low blood pressure may encompass inadequate cerebral perfusion and typically reflects poorer physiological status and hemodynamic instability, which could serve as a critical indicator for reconsidering life-sustaining measures.^25^

On the other hand, this study observed differences among three disease severity tools (CCI, SOFA, and SAPS II). While all three tools were significantly associated with code status transitions in univariate analysis, only SAPS II maintained statistical significance after multivariate analysis, potentially revealing inherent relationships or differences between them. SOFA primarily evaluates the functional status of six organ systems and was originally designed for sepsis patients.^26^ In aSAH, the severity of neurological injury, age factors, and overall physiological changes may be more representative than simple organ dysfunction.^19^ Previous studies have also confirmed difficulties in applying SOFA to sepsis in neurological disease patients.^26,27^ CCI predicts mortality through 19 different comorbidities,^28^ and despite demonstrating good predictive value in most hospitalized patients,^29^ it has limited prognostic value for patients with aSAH.^18,30^ This may reflect the uniqueness of aSAH: acute mortality risk primarily stems from primary brain injury, secondary complications (i.e., rebleeding, cerebral vasospasm, cerebral edema, increased intracranial pressure, and electrolyte imbalances), and hypertension,^31^ rather than long-term comorbidities. In contrast, SAPS II encompasses multiple physiological parameters, and previous research has demonstrated its adequate value in predicting mortality in patients with aSAH.^32^ Although the HR of SAPS II in multivariate analysis appears relatively small (HR = 1.018, *p* = 0.046), considering its scoring range of 0–163 points, this implies significant clinical importance—each one-point increase raises the risk of code status transition by 1.8%, which may explain SAPS II’s unique advantage in predicting code transitions in patients with aSAH.

We observed that glucose, BUN, and platelet levels in laboratory tests showed high significance (*p* < 0.001) in univariate analysis but did not maintain significance in multivariate analysis. Although previous studies have confirmed these parameters^33–35^ as important predictors of prognosis or severity in patients with aSAH, this discrepancy may reveal the unique nature of code status decision making. In clinical practice, individual laboratory abnormalities may be viewed as markers of disease progression but may not necessarily directly prompt the medical team and family to consider code status transitions. In contrast, changes in vital signs (such as decreased MBP) and disease severity (such as SAPS II) may be viewed as more direct and comprehensible prognostic indicators, thus playing a more critical role in code status discussions.

Hospice services emerged as the strongest independent predictor of code status transitions (HR = 6.951, 95% CI: 4.626–10.446, *p* < 0.001). After controlling for other factors including age, physiological parameters, and disease severity, hospice services were associated with nearly a seven-fold increase in the occurrence of code status transitions, highlighting the critical role of palliative medicine in the clinical decision-making process for patients with aSAH. The involvement of hospice services may play multiple roles in code status decisions. First, palliative teams typically excel at facilitating complex conversations about end-of-life care, communicating prognostic information in ways families can understand, and helping families clarify patients’ values and preferences. When confronting sudden crises like aSAH, this specialized communication support may be crucial for families making decisions to limit life-sustaining measures.^36,37^ Second, hospice services themselves may reflect the medical team’s assessment of patient prognosis, with their involvement potentially indicating that clinical practice has begun to consider terminal care goals rather than purely curative ones. However, due to the retrospective design of this study, we cannot determine the exact temporal relationship or causal direction between palliative services and code status transitions. The involvement of palliative services might be a result of the clinical team identifying a potentially poor prognosis, a factor prompting reassessment of code status, or initiated after limitations of life support were already being considered. Therefore, even though the findings echo that patients with aSAH who received hospice services typically represent a population with more severe illness and higher mortality,^38^ this association should be interpreted cautiously.

## Limitations

This study has several limitations: First, as the MIMIC-IV database is primarily based on ICD codes and physiological parameters, we cannot determine the exact temporal relationships or causal directions of certain clinical interventions, such as the timing of hospice service involvement. Second, important clinical interactions such as family meetings, comfort care measures, and end-of-life care processes could not be included in our analysis scope as they cannot be retrieved through ICD codes. Third, although the database recorded the code status of all patients during hospitalization, due to limitations of the ICD coding system, we cannot identify which health care team members participated in the code status discussion process. Finally, we excluded patients who did not survive, patients who already had advance health care directives, and patients with no code status records.

## Conclusion

This study examines factors associated with code status transitions in ICU patients with aSAH, finding that 25.8% (*n* = 146) of initially full-code patients transitioned to DNR/DNI during hospitalization. Cox regression analysis identified age, MBP, SAPS II, and hospice services as key predictors, which can help health care teams identify high-risk patients needing timely code status discussions. These findings emphasize the importance of periodic reassessment and hospice integration to ensure treatment plans align with patients’ values and prognostic realities, while also improving quality of life and reducing psychological stress for family members.^36,37^

## Abbreviations Used

AHA: the American Heart Association
aSAH: aneurysmal subarachnoid hemorrhage
BIDMC: the Beth Israel Deaconess Medical Center
BUN: blood urea nitrogen
CCI: Charlson comorbidity index
CI: confidence intervals
DNI: do-not-intubate
DNR: do-not-resuscitate
EHR: the electronic health records
EVD: external ventricular drainage
GCS: Glasgow Coma Scale
HR: hazard ratios
ICD: the International Classification of Diseases
ICH: intracerebral hemorrhage
ICU: intensive care unit
INR: international normalized ratio
MBP: mean blood pressure
MIMIC-IV: the Medical Information Mart for Intensive Care IV
RBC: red blood cell
SAH: subarachnoid hemorrhage
SAPS II: the simplified acute physiology score II
SOFA: Sequential Organ Failure Assessment
SPO_2_: peripheral oxygen saturation
WBC: white blood cell count
