# *Corrigendum to:* “Code Status Transitions of Patients with Aneurysmal Subarachnoid Hemorrhage in the Intensive Care Unit”

**DOI:** 10.1177/26892820251377757

**Published:** 2025-09-25

Su M-I, Hsiao C-Y, Ma J-C, Chang C-M. Code status transitions of patients with aneurysmal subarachnoid hemorrhage in the intensive care unit. *Palliative Medicine Reports* 2025;6(1):324–332; DOI: 10.1089/pmr.2025.0015.

In Figure 1, the patient flowchart inadvertently showed the screening process from the initial draft version prior to incorporating the reviewers’ suggestions to reset the case selection criteria, which reported incorrect patient numbers that were inconsistent with the data presented in Table 1 and the Results section of the article.

**FIG. 1.**
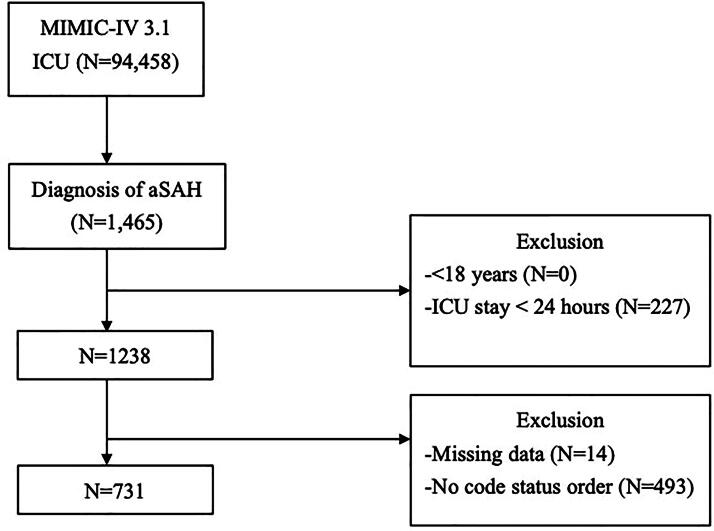
Flow diagram of study participants.

The authors have corrected the flowchart to accurately reflect the final sample size of 731 patients with aneurysmal subarachnoid hemorrhage (aSAH) that was consistently reported throughout the article text and tables. The corrected Figure 1 below now properly shows the database screening process that resulted in the inclusion of 731 cases for final analysis.

The journal editor confirmed that the changes to the flowchart do not alter the results or change the conclusions that can be drawn from this article, as the corrected figure now accurately represents the data that was analyzed and reported in the original article.

